# The toxicological mechanisms and detoxification of depleted uranium exposure

**DOI:** 10.1186/s12199-018-0706-3

**Published:** 2018-05-16

**Authors:** Yong-Chao Yue, Ming-Hua Li, Hai-Bo Wang, Bang-Le Zhang, Wei He

**Affiliations:** 1 Department of Chemistry, School of Pharmacy, Fourth Military Medical University, No. 169 Changle West Road, Xi’an, 710032 Shaanxi People’s Republic of China; 2 Department of Pharmaceutics, School of Pharmacy, Fourth Military Medical University, No. 169 Changle West Road, Xi’an, 710032 Shaanxi People’s Republic of China

**Keywords:** Depleted uranium, Toxicity, Decorporation, Chelating agents

## Abstract

Depleted uranium (DU) has been widely applied in industrial and military activities, and is often obtained from producing fuel for nuclear reactors. DU may be released into the environment, polluting air, soil, and water, and is considered to exert both radiological and chemical toxicity. In humans and animals, DU can induce multiple health effects, such as renal tubular necrosis and bone malignancies. This review summarizes the known information on DU’s routes of entry, mechanisms of toxicity, and health effects. In addition, we survey the chelating agents used in ameliorating DU toxicity.

## Background

Depleted uranium (DU) is uranium that contains less of the fissile isotope ^235^U than natural uranium. The isotopic composition of DU is typically 99.977% ^238^U, 0.2% ^235^U, and 0.0008976% ^234^U. DU is what remains after removal of enriched uranium, and may also be generated from the reprocessing of spent nuclear reactor fuel [1]. DU is known to exert both radioactive and chemical toxicity [2]. The radioactivity of DU is approximately 60% that of natural uranium, and their chemical properties are similar. Because of its low price, high penetrability, and pyrophoricity, DU has been widely used in both military and civilian activities [3]. At high temperatures, it can destroy armored and fortified structures and vehicles.

DU may be released into the environment as it is mined, processed, and applied. East Germany and Czechoslovakia released one billion tons of uranium-mined ores and residues into soil and surface waters between 1945 and 1989 [4]. NATO forces used DU weapons against Serbian heavy infantry in the Kosovo conflict in 1999, and more than 9 tons of DU was used in the war, raising concerns worldwide [5]. DU weapons have also been in other wars, including the Persian Gulf War and the Balkans conflicts. The physical half-life of DU exceeds 4.49 × 10^9^ years, and it can remain in soil and groundwater a long time, affecting local ecosystems [3].

DU enters the body via inhalation, ingestion, or dermal contact, damaging tissue. Both acute and chronic exposures can produce adverse effects, but chemical toxicity mainly ensues from acute exposure, and the kidney is its most vulnerable target. Renal DU toxicity is characterized by damage to the proximal tubulares, potentially leading to tubular necrosis [6]. Injections of 0.5, 1 and 2 mg/kg, DU in rats have been shown to damage renal function and mitochondria [7]. Intragastric DU administered to rats (204 mg/kg) modulated the expression of cytochrome enzymes involved in vitamin D metabolism in the liver and kidney [8]. Chronic DU exposure can also affect the function of multiple tissues and organs such as the kidney [9], bone [10], brain [11], and reproductive systems [12]. Rodents exhibited testicular histopathological abnormalities and decreases in pregnancy rates and spermatid numbers after a chronic dose of 10–80 mg/kg/day uranium [13, 14]. Armant et al. [15] demonstrated that chronic parental exposure to 20 μg/L DU could impair the histological ultrastructure of organs and molecular development in zebrafish progeny.

This review summarizes the data available on DU toxicity and compounds used in its detoxification.

## Entry routes and health effects

### Entry routes

Understanding the absorption and biodistribution of DU is necessary to better prevent and mitigate its toxic effects. The factors that affect the absorption and bioavailability of DU are complex and include its solubility, physicochemical form, and entry route. The more soluble forms, such as (NH_4_)_4_UO_2_(CO_3_)_3_ and UO_2_(NO_3_)_2_, can diffuse more easily in body fluids. Less soluble forms, such as (NH_4_)_2_U_2_O_7_, UO_2_(CH_3_COO)_2_, and UO_2_ tend to accumulate in specific organs and cause local toxicity [16]. Uranium in surface water can be present as free metal ions or as complexes with inorganic ligands (e.g., phosphates and carbonates) or humic substances. Different uranium species can also interact. UO_2_^2+^ and UO_2_OH^+^ are the forms typically available to organisms, but the inorganic ligands and humic substances involved can reduce the activity of UO_2_^2+^ and UO_2_OH^+^, lowering their bioavailability [17].

**Fig. 1:**
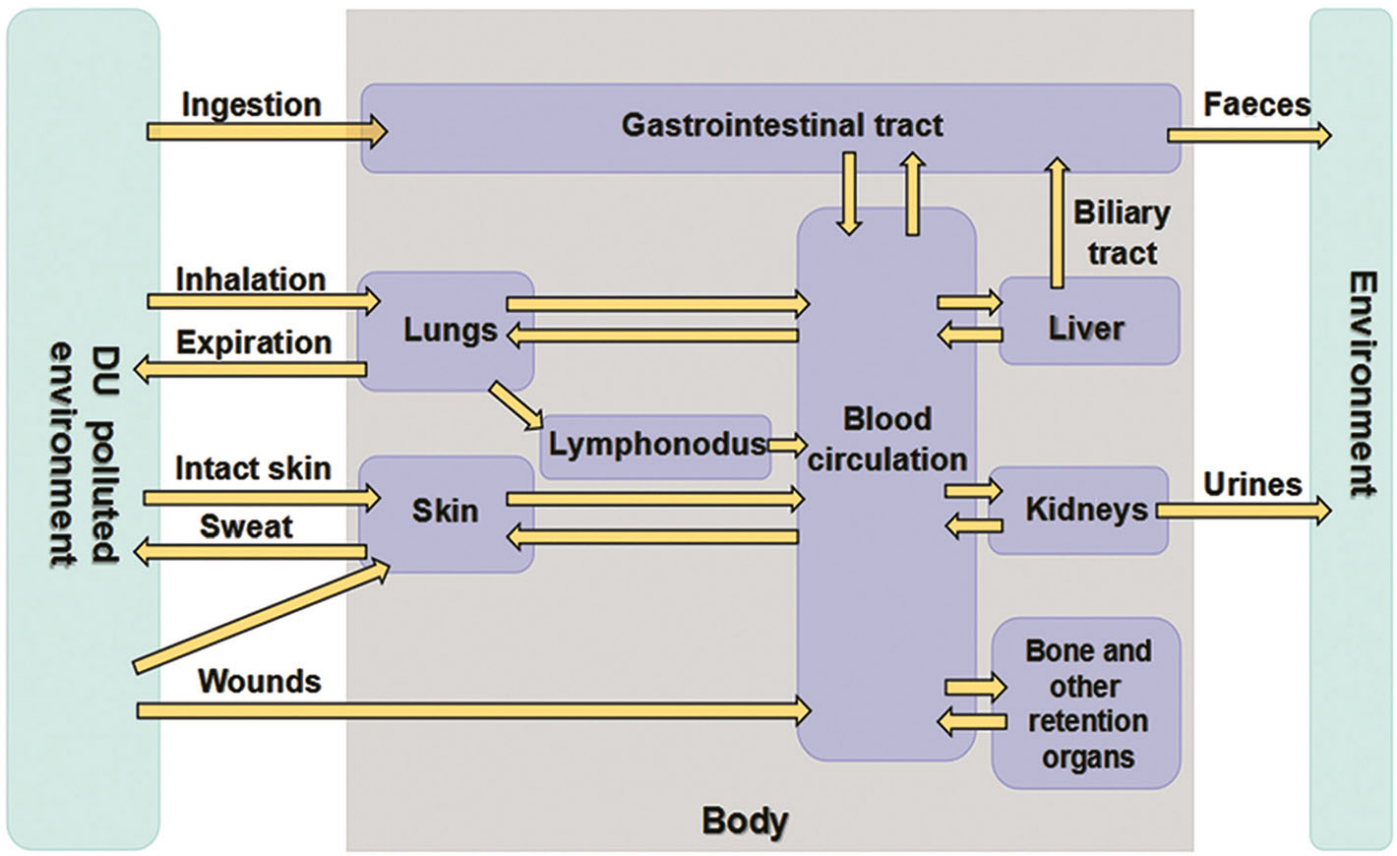
Biokinetic process of DU contamination

DU exposure sources include inhaled aerosols, ingestion of DU-contaminated food and water, and dermal penetration through intact or broken skin [3]. Figure 1 shows the biokinetics of DU. Once DU enters systemic circulation, it can be excreted through urine, feces, sweat, and exhaled breath, but some is deposited in sensitive organs and tissues, eventually interacting with cellular structures and impairing their normal functions.

Respiration is considered to be the major mode of exposure. DU aerosols can be generated through industrial activities or detonation of DU-containing weapons and dispersed in atmosphere. The concentration, shape, and particle size of inhaled DU particles can affect their absorption. DU nanoparticles less than 100 nm in diameter have been shown to be rapidly absorbed and deposited in the respiratory tract in rats [18]. DU particles can penetrate deep into lung alveoli and dissolve rapidly in blood, while the mucociliary escalator can transport particles to the mouth, from which they enter the gastrointestinal tract [16].

Ingestion is considered an unlikely exposure route in industrial settings, as it can be controlled with safety regulations. It may, however, be more common in civilians and soldiers in DU-polluted war zones [19]. DU absorption in the gastrointestinal tract is low, and has been measured in hamsters as 0.11% for uranium dioxide and 0.8% for uranyl nitrate [20]. In humans, this value is approximately 2% for soluble uranium and 0.2% for relatively poorly soluble tetravalent compounds, such as UF_4_, UO_2_, and U_3_O_8_ [21].

Dermal penetration is a common route of exposure, especially through broken skin, that comes into contact with DU aerosols and contaminated surfaces. DU deposited on skin can reach systemic circulation and spread throughout the body. DU was detected in the muscles and kidneys of rats after 6 h of dermal treatment with UO_2_(NO_3_)_2_. After 24 h, the absorption rate of uranium through intact and excoriated skin was approximately 0.4 and 38%, respectively [22]. DU skin adsorption, however, can be affected by factors, such as the solubility, the exposure duration or area, and other physiological and physical parameters [16].

Regardless of entry route, DU will enter general circulation and bind to target organs. After intravenous injection, approximately 50% of DU is excreted in the urine, 25% can accumulate in the bone, and the remaining 25% in the soft tissues [23]. Urinary excretion accounts for 60–86% of the absorbed dose, while 1–2% of DU is removed through feces [16].

### Health effects

Uranium toxicity has been investigated extensively. DU is known to induce genomic instability such as DNA double-strand breaks, chromosome aberrations and micronuclei formation [24], and to exert adverse effects on organs such as the kidney [25], bone [26], and brain [27]. Lung cancer [28] and lymphoma [29] are also thought to be related to DU over-exposure.

The distinction between DU’s radiotoxicity and chemical toxicity is not well defined [30]. The main toxic mechanism of DU appears to be the generation of oxidative stress and reactive oxygen species (ROS) through a reduction in cellular free-radical scavengers and antioxidants. An increase in ROS production and suppression of antioxidant enzyme activities [31].

### Renal toxicity

The kidney is considered the organ most vulnerable to soluble DU compounds. Absorbed uranium is filtered through the glomerulus and is then bound as UO^2+^ to anionic sites of the epithelial brush border in the proximal tubules [32]. DU may penetrate the proximal tubule through the type IIa sodium-dependent phosphate co-transporters [33] and/or endocytosis [34, 35]. Intracellular DU can disrupt the electron transfer chain, leading to ROS formation, lipid peroxidation, glutathione oxidation, and subsequent mitochondrial damage in proximal tubules [36].

Acute over-exposure to DU in humans is rare, but studies in laboratory animals have shown that the toxic threshold of single-dose intraperitoneal DU treatment was approximately 0.5 mg/kg [25]. Regardless of exposure routes and animal species, single-dose exposures (> 2 mg/kg) are nephrotoxic and sufficient to alter the biochemical parameters of renal function (blood urea nitrogen, plasma creatinine, N-acetyl glucosaminidase, and alkaline phosphatase) [25]. Chronic exposure can occur in DU-polluted environments, but the relationship between DU exposure and renal damage is still unclear, as chronic nephritis, like most renal impairments, gradually develops into irreversible damage and may not be induced by exposure to DU [37]. Animal experiments have been inconclusive. A chronic DU dose of 0.02 mg/kg resulted in renal alterations in rats [38]. While 30 mg/kg DU administered over 3 months did not induce biochemical changes in the rabbits [39].

### Bone toxicity

Bone is known to accumulate uranium over long periods, and growing bone surfaces are a major target. DU shortens bones by altering the structure of the trabecular zone, promoting bone resorption, and inhibiting bone formation [26]. In both humans and animals, and especially in the young, uranium bone deposition has been shown to be time- and dose-dependent. This has been linked to the high affinity between uranium and phosphate anions, resulting in UO_2_^2+^ replacing calcium cations [40]. The elimination half-life of uranium from bone has been estimated at 70–200 days [26]. Basset et al. [41] have suggested that the accumulation of uranium in bone may be related to the fetuin-A protein, which has a high affinity with uranium and is major carrier of uranium in blood, but is also involved in bone mineralization.

Bourgeois et al. [40] studied the influence of uranium on rat femur and reported that uranium was preferentially transported to calcifying zones after exposure and subsequently accumulated in the calcifying cartilage, the periosteal and endosteal areas of femoral metaphysis, and newly formed bone tissue along trabecular bone. High accumulation was also found in micro-vessels and bone trabeculae.

Furthermore, uranium may alter the metabolism of vitamin D and affect normal bone functions and growth indirectly. Uranium-induced alterations in vitamin D production and levels may modify mineral homeostasis, affect bone maintenance, and reduce bone growth in the elderly [42].

### Hepatotoxicity

DU enters the bloodstream rapidly after exposure, but little is retained in the liver, a major organ for the storage and detoxification of heavy metals [43]. No clear histological alterations have been observed in the livers of DU-exposed rats, although the levels of alanine aminotransferase and aspartate aminotransferase did increase following a chronic exposure to DU through drinking water [44]. Yapar et al. [45] found a significant decrease in reduced glutathione (GSH) levels and an increase in serum alanine aminotransferase, aspartate aminotransferase, and malondialdehyde in DU-treated mice. Pourahmad et al. [46] reported increased ROS formation and GSH depletion in isolated hepatocytes following exposure to uranyl acetate. DU-induced mitochondrial dysfunction and uncoupling of oxidative phosphorylation may contribute to hepatic cell death and subsequent clinical complications.

### Lung toxicity

Respiration is considered the major route of DU exposure. Inhalation of DU aerosols, especially insoluble DU aerosols retained in lung tissue and nearby lymph nodes, can cause damage, such as emphysema and pulmonary fibrosis, and may lead to lung cancer.

Petitot et al. [18] demonstrated that approximately 26.2% of DU particles can deposit in the lung after inhalation. Approximately one-fifth of deposited particles are rapidly cleared to extrapulmonary organs, while the remaining particles are cleared in situ, and the retention half-life is approximately 141.5 days. Larger DU particles are typically deposited in the upper respiratory tract, while particles less than 10 μm in diameter are deposited in the bronchi and alveoli. Some DU particles can penetrate deep into lung alveoli and dissolve in the blood, but most remain in the lungs, and the mucociliary escalator may also transport these particles to the mouth, where they enter the gastrointestinal tract.

Periyakaruppan et al. [47] evaluated uranium toxicity in rat lung epithelial cells and found that exposure resulted in oxidative stress and decreases in antioxidant activity and proliferation. Xie et al. [48] reported that DU exposure led to anchorage-independent growth and loss of contact inhibition in human bronchial epithelial cells, as well as chromosome instability and a neoplastic phenotype.

### Neurotoxicity

The brain is a target organ of heavy metals such as manganese, mercury, zinc, and lead. DU may also impair cerebral functions, but how DU enters and accumulates in the brain is unclear. Lemercier et al. [49] concluded that uranium does not damage the blood-brain barrier in rats. Tournier et al. [27] demonstrated that uranium could be transported directly from the nasal cavity to the olfactory bulb in rats after inhalation or instillation. Uranium may cause behavioral changes and affect the circadian rhythm, locomotion, and cognitive functions in [11]. Chronic DU exposure has been shown to affect the genetic pathway involved in visual perception in zebrafish and to modify the transcriptomic pattern in this brain area [15]. In humans, however, a relationship between DU exposure and behavior changes has not been established. Gulf war syndrome may be related to DU exposure or to combat stress. Therefore, the neurotoxicity of DU requires further research.

### Immunotoxicity

The immune system is also sensitive to chronic DU exposure [50], which may result in autoimmune disease, infectious diseases, and cancer. Multiple studies have confirmed that immune cells are affected by DU. Kalinich et al. [51] determined that macrophages can absorb uranium in a time-dependent manner, leading to apoptosis. Wan et al. [52] demonstrated that DU can induce damage to splenic CD4^+^ T-cells and peritoneal macrophages in a dose-dependent manner. Furthermore, a non-cytotoxic DU dose may damage the immune functions by modulating the expression of genes, involving interleukin activity, signal transduction, neurotrophic factors, chemokines, and chemokine receptors.

Information on DU immunotoxicity in animals is sparse. Hao et al. [53] showed that a high dose (300 mg/kg) of DU could significantly inhibit immune function in Kunming mice, probably owing to the instability in T helper 1 and 2 cytokines, ROS imbalances have also been linked to DU immunotoxicity in zebrafish [54].

### Radiotoxicity

Compared with natural uranium, DU has low radioactivity and is not considered to exert significant radiotoxicity. Studies of animals and occupationally exposed individuals have demonstrated that the health effects of DU are mainly attributable to chemical toxicity; however, Miller et al. [55] showed that the radioactivity of DU contributed to its biotoxicity. The dicentric frequency of human osteoblast cells was significantly elevated in vitro following a 24 h exposure to 50 M DU, in contrast with the effects of the radiation-free heavy metals nickel and tungsten. The neoplastic transformation frequency also increased. The same group recently found that DU exposure reduced cell survival and increased neoplastic transformation, perhaps owing to radiotoxicity [2].

In summary, DU enters the body via ingestion, inhalation, and dermal contact, and can exert both chemical and radiological damage. DU can impair the normal functions of the kidney, bone, liver, lung, brain, and immune system, and reducing DU damage requires effective therapeutic measures.

## DU detoxification

DU accumulation may be reduced by increasing its elimination or decreasing its absorption and distribution. Chelating agents and other chemicals are used to eliminate DU and reduce the risk of toxicity.

### Chelating agents

Chelating agents have been used extensively to treat acute and chronic human intoxication to a wide range of metals [56]. The formation of soluble chelating agents may reduce DU deposition in organs and accelerate its elimination. Effective candidate chelating agents must possess several characteristics [57]. First, the compound should be an efficient scavenger of DU via deprotonation under physiological conditions. Second, the agent’s site of action should be suitable for eliminating the metal. Third, the chelating agent should be selective for DU and not eliminate necessary trace elements. Fourth, the chelating agent should be lipophilic, highly bioavailable, and possess low toxicity. Fifth, the agent, once bound to DU, must be excreted effectively. Sixth, the agent must be easily acquired at low cost and be administered orally.

**Fig. 2:**
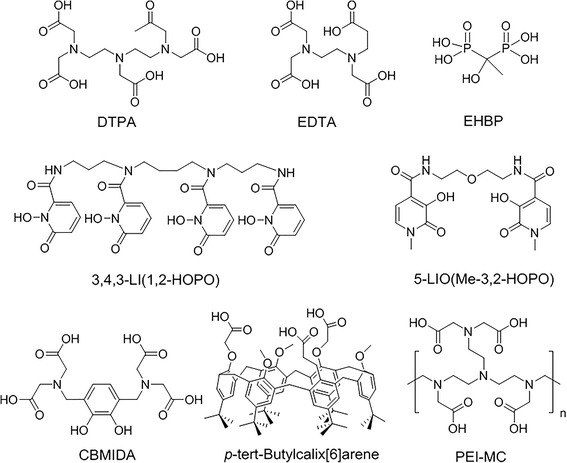
The chemical structure of chelating agents for DU

Several categories of chelating agents have been synthesized and studied for their DU decorporation efficiency in vivo. Agents with poor tissue specificity and high toxicity have been modified to obtain satisfactory results. Figure 2 displays the chemical structures of the most important chelating agents.

### Polyaminocarboxylic acids

Polyamino carboxylic acids, the most representative chelating agents, consist of diethylenetriaminepentaacetic acid (pentetic acid, DTPA) and ethylenediaminetetraacetic acid (EDTA), the original ligands used for decorporating uranium in vivo. While EDTA can bind to multiple metals, uranium (VI) and EDTA complexes are unstable over pH 7.5 and break up into free EDTA and a diuranate precipitate [58]. DTPA was considered the gold standard in chelating uranium in vivo and often served as a positive control. Pharmacokinetic studies of ^14^C-DTPA have shown that DTPA is not metabolized and cleared by glomerular filtration [59, 60], but required prompt administration parenterally (intravenous or subcutaneous treatment). Side effects such as nephrotoxicity, teratogenicity, embryotoxicity, and suppressed hematopoiesis have been reported for DTPA [16]. To inhibit its effects on the normal biological functions of essential trace metals, DTPA was reformulated as either the calcium or zinc chelate. Ca-DTPA could replace calcium and chelate metal ions [61], but exhibited poor selectivity and affinity for DU under physiological conditions, as well as an increased risk of acute nephritis because of uranium deposition in the kidney. The complexes Ca-DTPA formed with DU were unstable, rendering it unsuitable for chelating DU. Zn-DTPA was also not satisfactory for decorporating DU [61, 62].

### Siderophores

Siderophores such as catechoylamide (CAM) and hydroxypyridones (HOPO) are low molecular weight but highly selective chelating agents. Siderophores include catecholate, hydroxamate, and carboxylic acid functional groups that can bind to a variety of metals. Within the CAM family, the efficacy of catechol-3,6-bis(methyliminodiacetic acid) (CBMIDA) as a DU chelator has been shown, particularly when administered as an intramuscular injection [63]. Fukuda et al. [64] compared the oral and parenteral activity of CBMIDA in rats and found that they were similar. Furthermore, CBMIDA was superior to DTPA in eliminating DU.

Durbin et al. [65] tested 10 siderophores as chelators of UO_2_^2+^ in vivo and selected 5-LIO(Me-3,2-HOPO) for its low toxicity, high efficacy, and affordability. Kullgren et al. [66], however, concluded that only 3,4,3-LI(1,2-HOPO) could form stable complexes with UO_2_^2+^ to increase uranium excretion significantly. Choi et al. [67] evaluated the properties of 3,4,3-LI(1,2-HOPO) in plasma and microsomal and gastrointestinal fluids using the Caco-2 colorectal adenocarcinoma cell line. They found that 3,4,3-LI(1,2-HOPO) was not affected by hepatic cytochrome P450 metabolism and remained stable at 37 °C after 1 h. In 2014, 3,4,3-LI(1,2-HOPO) received approval by the US Food and Drug Administration for phase I clinical trials [16].

### Polyphosphonates

Ethane-1-hydroxy-1,1-bisphosphonate (EHBP) is the most representative polyphosphonate chelating agent. EHBP is highly specific for bone, owing to its strong chemical affinity with the surface of calcium hydroxyapatite [68]. As EHBP can reduce the bone turnover rate and inhibit bone resorption, it has been widely used to treat osteopenic diseases and Paget’s disease under the name Didronel®. EHBP has also been shown to be effective in inhibiting bone formation after acute uranium exposure and counteracting the effect of lethal doses of UO_2_(NO_3_)_2_ in young rats [69]. Henge-Napoli et al. [70] showed that intramuscular administration of EHBP can reduce the accumulation of uranium in rats and exert renoprotective effects even if treatment was postponed 30 min after uranium exposure. Furthermore, EHBP was shown to reduce renal lesions and the lethal effect of DU in mice after oral administration [71]. EHBP has long been used in clinical settings and its effects have been well-studied, so it may be an effective DU chelating agent.

### Calixarenes

Calixarenes are macrocyclic ligands, consisting of phenolic units and linked by methylene bridges at their ortho positions, and the hydroxy functionalities of these ligands can form coordination complexes with several metals simultaneously [72]. Calixarenes have exhibited good affinity and selectivity for several metals, and have been considered as complexing agents for detecting radioactive elements such as plutonium and uranium in urine or the environment [73].

**Fig. 3:**
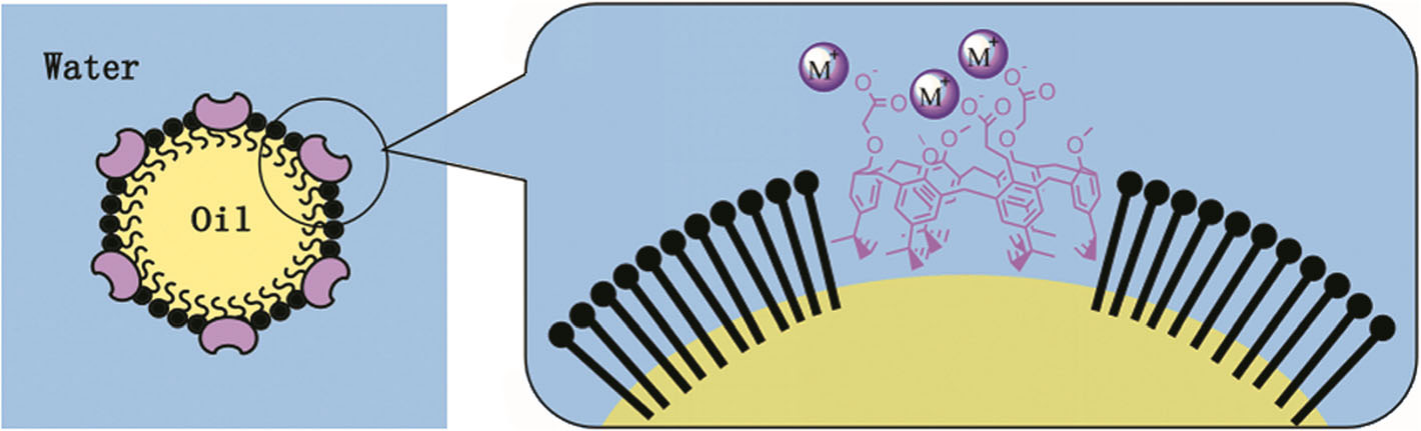
The structure of calixarene nanoemulsion

The compound *p*-tert-butylcalix[6]arene, which has three carboxylic groups, has shown a high affinity with UO_2_^2+^ [74]. As there was no specific and efficient therapeutic measure for uranium skin contamination, Spagnul et al. [75] developed an oil-in-water nanoemulsion delivery system for *p*-tert-butylcalix[6]arene (Fig. 3). The calixarene nanoemulsion had a uranium clearance rate of approximately 80% from aqueous-contaminated solution, and 98 and 97% from intact and injured pig ear skin explants, respectively [76]. And the same group later demonstrated the scope of decontamination activity on injured skin, which was approximately 92–94% with no overt side effects [77]. This calixarene nanoemulsion may be a potent chelating agent for DU skin decontamination.

### Methyl-carboxylated poly(ethylenimine)

Unlike molecular chelating agents, macromolecules are a relatively recent addition to uranium decorporation strategies. Macromolecules have multiple chelating sites per area unit and a high affinity for their targets. The biodistribution of macromolecules depends on their size, modulating their permeability and retention. In addition, macromolecules, especially highly soluble polymers, have been studied extensively and can easily be functionalized [78]. The functionalized methyl-carboxylated poly(ethylenimine) (PEI-MC) has been used as an effective scavenger of heavy metals from contaminated water [79]. Lahrouch et al. [78] assessed PEI-MC as a uranium decorporation agent under physiological pH conditions and found that the maximum load of uranium (VI) was 0.47 mg per milligram of PEI-MC, a far better load than those of sodium bicarbonate and Ca-DTPA.

## Other decorporation drug

### Sodium bicarbonate

Sodium bicarbonate has long been used to chelate DU, which is administered by slow intravenous infusion or orally until urinary pH levels reach 8.0–9.0 [80]. Sodium bicarbonate is believed to increase the number of bicarbonate ions in blood and the pH in the proximal tubules. The uranyl ion can more easily form a complex with bicarbonate, which is regarded as less nephrotoxic and more stable in vivo, and is filtered promptly by the kidneys. Ohmachi et al. [80] demonstrated the renoprotective effect and uranium chelation efficacy of sodium bicarbonate in a rat model, but the main drawbacks of sodium bicarbonate are its low efficiency and high acid-base disturbance. Fukuda et al. [81] combined sodium bicarbonate and other chelating agents in rats, with mixed results.

### Zinc and metallothioneins

Zinc is an essential trace element required for the normal function of cells, and can inhibit DU-induced apoptosis [82]. Metallothionein is a sulfur-containing protein with low molecular weight, which is widely distributed in tissues and organs. Two of its human isoforms have been shown to be involved in the detoxification of heavy metals, perhaps by reducing the levels of oxidative stress and apoptosis and by upregulating the expression of sodium glucose co-transporters [83].

Hao et al. [84] demonstrated that DU detoxification and survival rates in rats were notably improved by pretreatment with zinc, probably because zinc induced metallothioneins. Compared with effects in wild-type mice, pronounced renal dysfunction and morphological damage in metallothionein-null mice have been shown following DU administration [85]. These findings suggest that zinc and metallothioneins may be beneficial in preventing and treating DU-induced nephrotoxicity, but more studies are needed before clinical application.

### Hydrogen sulfide

Hydrogen sulfide is a toxic gas that can damage the respiratory and nervous systems. Endogenous hydrogen sulfide has been identified as a signal molecule of nitric oxide and carbon monoxide, and is produced from cysteine or homocysteine by the action of cystathionine *β*-synthase, cystathionine *γ*-lyase, and 3-mercaptopyruvate sulfurtransferase along with cysteine aminotransferase [86]. Hydrogen sulfide is anti-inflammatory, anti-oxidative, and cytoprotective [87, 88], and hydrogen sulfide supplementation may protect organs against DU toxicity.

Zheng et al. [89] found that the generation of endogenous hydrogen sulfide was downregulated in rat kidney following exposure to uranium. Treatment with sodium hydrosulfide (28 or 56 mmol/kg/day) increased hydrogen sulfide to protective levels by activating the NF-E2-related factor 2 pathway and reducing inflammatory responses. These results indicate that hydrogen sulfide can protect against uranium-induced nephrotoxicity.

## Conclusion

DU has been widely applied in the nuclear industry and military activities, but its release into air, soil, and water can adversely affect organisms and ecosystems. DU enters the body by inhalation, ingestion, or dermal contact, and can impair the normal function of the kidney, bone, liver, and brain. However, DU spillings in the environment are expected to increase due to the increased demand in nuclear fuel demand.

Multiple compounds have been synthesized and tested for their suitability to chelate DU, but many exhibit poor tissue specificity and high toxicity, precluding clinical application. 3,4,3-LI(1,2-HOPO) exhibits low acute toxicity in mice, is well-tolerated at high doses in rats, and shows good oral bioavailability. This is the most promising chelation agent and may be a candidate for clinical trials. Other compounds have shown good efficacy but are still in the preclinical phase. Drug combinations, metered-dose inhalers, and novel drug-delivery systems such as liposomes should also be considered, as they may be more efficient than current conventional therapies.

## Abbreviations

CAM: Catechoylamide
CBMIDA: Catechol-3,6-bismethyliminodiacetic acid
DTPA: Diethylenetriaminepentaacetic acid
DU: Depleted uranium
EDTA: Ethylenediaminetetraacetic acid
EHBP: Ethane-1-hydroxy-1,1-bisphosphonate
GSH: Glutathione
HOPO: Hydroxypyridones
PEI-MC: Methyl-carboxylated poly(ethylenimine)
ROS: Reactive oxygen species
